# Epigenetic Factors of Disease

**DOI:** 10.3390/diseases7020042

**Published:** 2019-06-14

**Authors:** Ilham Alshiraihi, Mark A. Brown

**Affiliations:** 1 Cell and Molecular Biology Program, Colorado State University, Fort Collins, CO 80523, USA; Ilham.Alshiraihi@rams.colostate.edu; 2 Department of Clinical Sciences, Colorado State University, Fort Collins, CO 80523, USA; 3 Epidemiology Section, Colorado School of Public Health, Fort Collins, CO 80523, USA; 4 Department of Ethnic Studies, Colorado State University, Fort Collins, CO 80523, USA

**Keywords:** epigenetics, post-translational modifications, chromatin, nucleosome remodeling

## Abstract

The development of tissues involves the direction of specific programs for gene expression among distinct cell types. These programs are often established in a heritable state by virtue of epigenetic mechanisms and corresponding pathways of cellular memory. Thus, the broad synchronization in patterns of gene expression ultimately dictates cellular consequences. Aberrations in these epigenetic mechanisms are known to be associated with a range of diseases. Herein, we highlight epigenetic factors that, when aberrantly expressed, lead to a broad range of diseases. Further, we call upon the community of biomedical researchers to share their findings related to the epigenetic factors of disease.

## 1. Introduction

The association between aberrations of epigenetic factors and disease highlights the significance of understanding epigenetic mechanisms. Such mechanisms normally function in the global synchronization of patterns in gene expression [1]. The basis for such gene regulation resides in cellular pathways that guide the packaging of DNA into chromatin, thereby, regulating the scale of accessibility to transcription complexes [2,3]. Aberrations in such epigenetic pathways are associated with a range of pathological outcomes [4,5,6,7,8]. 

## 2. Discussion and Conclusions

Key pathways of epigenetic regulation include the following: DNA methylation; histone modifications; ATP-dependent nucleosome remodeling; and non-coding RNA (Table 1).

DNA methylation at cytosines typically occurs in CpG-rich regions and, ultimately, affects DNA–protein interactions. These, in turn, can affect the recruitment of chromatin-modifying complexes and transcriptional complexes. Changes in the recruitment of these complexes are known to impact a broad range of conditions including embryonic development, epigenetic inheritance, genomic stability, allele-specific expression, inactivation of the X chromosome, and other biological processes [9,10].

Histone modifications are commonly manifested through post-translational events in histone tails. Common modifications include acetylation, methylation, and phosphorylation. Such changes to histone tails impact cellular processes such as gene expression, cell cycle progression, and DNA replication/repair by altering chromatin structure and/or the recruitment of regulatory complexes [11,12]. 

ATP-dependent chromatin modifications are facilitated by ATPase-domains of nucleosome remodeling complexes [13]. These modifications affect the level of chromatin accessibility to protein complexes. The downstream impacts of these complexes range from cell differentiation and cell cycle progression to gene expression and DNA replication/repair [14].

The non-coding RNA interaction with CpG islands of promoters is known to impact levels of gene expression [15,16]. This mechanism often occurs in tandem with DNA methylation-mediated regulation. The fact that these events often occur in a sequence-specific manner has facilitated the analyses of gene-specific impacts of non-coding RNA on both a temporal and spatial basis [17,18].

Aberrations in epigenetic mechanisms affect a broad range of physiological processes and often result in pathological conditions [19]. To date, a range of disease categories, such a neuropathological [5] and oncological [8], have been associated with missteps in epigenetic regulation. Despite great progress in our understanding of diseases related to epigenetic events, there remain major gaps in information about the interrelatedness of epigenetic factors that ultimately preclude most proposed clinical applications of epigenetic modulators. Thus, we call upon the community of biomedical researchers to share their findings related to the epigenetic factors of disease in this Special Issue of *Diseases* titled “Epigenetics and Disease.” For information on this Special Issue, visit https://www.mdpi.com/journal/diseases/special_issues/Epigenetics_Disease. 

## Figures and Tables

**Table 1:** Key pathways of epigenetic regulation.

Pathway	Mechanism of Action	Examples of Impacted Conditions
DNA Methylation	DNA–protein interactions	embryonic development; epigenetic inheritance; genomic stability; allele-specific expression; inactivation of the X chromosome;
Histone Modifications	post-translational modifications of histone tails	gene expression; cell cycle regulation; DNA replication; DNA repair; chromatin structure
ATP-Dependent Chromatin Modifications	chromatin remodeling complexes containing an ATPase domain	cell differentiation; gene expression; cell cycle regulation; DNA replication; DNA repair; chromatin structure
Non-Coding RNA	RNA-targeting of CpG islands; small interfering RNAs	gene expression
